# Reduced self-control leads to disregard of an unfamiliar behavioral option: an experimental approach to the study of neuroenhancement

**DOI:** 10.1186/1747-597X-8-41

**Published:** 2013-12-06

**Authors:** Wanja Wolff, Franz Baumgarten, Ralf Brand

**Affiliations:** 1 Department of Sport and Exercise Psychology, University of Potsdam, Am Neuen Palais 10, 14469 Potsdam, Germany

**Keywords:** *Neuroenhancement*, *Self-control*, *Ego-depletion*, *Prevention*

## Abstract

**Background:**

Neuroenhancement (NE), the use of psychoactive substances in order to enhance a healthy individual’s cognitive functioning from a proficient to an even higher level, is prevalent in student populations. According to the strength model of self-control, people fail to self-regulate and fall back on their dominant behavioral response when finite self-control resources are depleted. An experiment was conducted to test the hypothesis that ego-depletion will prevent students who are unfamiliar with NE from trying it.

**Findings:**

130 undergraduates, who denied having tried NE before (43% female, mean age = 22.76 ± 4.15 years old), were randomly assigned to either an ego-depletion or a control condition. The dependent variable was taking an “energy-stick” (a legal nutritional supplement, containing low doses of caffeine, taurine and vitamin B), offered as a potential means of enhancing performance on the bogus concentration task that followed. Logistic regression analysis showed that ego-depleted participants were three times less likely to take the substance, *OR* = 0.37, *p* = .01.

**Conclusion:**

This experiment found that trying NE for the first time was more likely if an individual’s cognitive capacities were not depleted. This means that mental exhaustion is not predictive for NE in students for whom NE is not the dominant response. Trying NE for the first time is therefore more likely to occur as a thoughtful attempt at self-regulation than as an automatic behavioral response in stressful situations. We therefore recommend targeting interventions at this inter-individual difference. Students without previous reinforcing NE experience should be provided with information about the possible negative health outcomes of NE. Reconfiguring structural aspects in the academic environment (e.g. lessening workloads) might help to deter current users.

## Background

Neuroenhancement (NE), the use of psychoactive substances to enhance one’s cognitive functioning without a medical indication to do so, seems to be on the rise [1-3]. Healthy individuals decide to take a substance in order to augment their cognitive capacity from a proficient level to even higher levels [4]. Researchers have proposed a behavioral instead of a substance-based approach of NE [5]. This approach holds that if an individual consumes a caffeinated synthetic drink (e.g. an “energy drink”, *lifestyle drug NE*) in the explicit expectation of increasing alertness, that individual is neuroenhancing. The same goal may be reached even more effectively with amphetamine derivatives (e.g. Ritalin; *prescription drug NE*), or with illicit drugs (e.g. cocaine; *illicit substance NE*). A substance’s actual effectiveness is of only limited relevance, for example when onset of a novel behavior is explained; it is the *assumed functionality* of a substance which is important to understanding the psychological roots of NE behavior [5].

The ethics and fairness of NE usage are currently the subject of controversy e.g. in neuroethics [6-8]. However, in the light of potential long-term negative effects on both mental and physical health one should not jump to the conclusion that NE is a reasonable and justifiable method of increasing individual performance [9].

Most of the published research on NE addresses epidemiological issues e.g. [1,10,11]. Recent studies indicate a growing prevalence of NE, especially among university students with an annual prevalence of about 17.1% in medicine students, and up to 25.4% in sports students as extreme examples [1]. An important step in deepening the understanding of NE is to investigate what causes people to start using neuroenhancers. A handful of studies have investigated possible psychological drivers of NE [5,8,12-14], for example global school-related distress and experiencing school demands as overwhelming have been shown statistically to predict NE [5,8]. It has also been suggested that students employ substances (e.g. painkillers) to cope with negative study-related outcomes [12]. Previous recommendations for prevention have therefore focused almost exclusively on stress as a major cause of NE among students [5,15]. There have as yet been no published experimental studies, and these are prerequisite for drawing more causal inferences about stress or other possible psychological factors in use of NE. The experimental approach to NE behavior presented here may suggest alternative starting points for prevention efforts.

Self-regulation theory provides the conceptual framework for this study [16]. According to this theory, individuals consume NE substances as a means of self-regulating their mental capacities and in order to enhance their performance. Our hypotheses were derived from the *strength model of self-control*[17]. We aimed to create an experimental setting, which tends to induce first use of NEs among undergraduate students who have never tried such a substance before.

The strength model of self-control suggests that resources for self-control can be depleted by mentally exhausting tasks. The resulting psychological state is *ego-depletion*[18]. Studies have shown that this state is associated with impaired cognitive processing, enforced impulsiveness, passivity and reduced motivation [19]. One of the strength model’s central predictions is that once finite self-control resources have been depleted, individuals will fall back on their *dominant behavioral response* i.e. behave according to habit. The effects of experimentally reduced self-control have been studied in various settings [20]. For example, in the state of ego-depletion participants were susceptible to drinking more alcohol and dieters were more likely to break their diet [20].

Most of these ego-depletion studies sought to explain why people fall back into an undesired or unhealthy behavior. This is different from the objective of our experiment. Students may perceive NE as a functional and legitimate means of achieving their academic goals. In fact, ethical standards relating to NE, e.g. whether it is fair or unfair to use a neuroenhancer in order to improve one’s performance in university exams, are currently in dispute [21]. NE is still an ambiguous behavior, and may be neither especially tempting nor repulsive to at least some individuals. From the perspective of the strength model ego-depletion is predictive for an individual’s dominant response to a given situation; this results in different predictions about NE for NE first-time and habitual users. Ego-depletion, as a state in which mental resources for self-regulation are minimal, will lead students for whom NE is a dominant response to take the substance, whilst potential first-time users will fall back on their dominant response: *not* taking the substance. Consuming a neuroenhancer may therefore represent to non-depleted first-time users a means of self-regulating (and vice versa for depleted habitual users). It is inconsistent with the strength model of self-control, to assume that high levels of stress or heavy workloads will automatically increase *all* students’ readiness to consume neuroenhancing substances. On the other hand, it is consistent with the theory to recognize individual differences based on the students’ habits (i.e. the experience they have already had with NE). This knowledge may prompt the design of more targeted interventions in the future.

The present study focused exclusively on students who reported having no previous experience with neuroenhancers as a means of improving cognitive performance. We investigated experimentally the hypothesis that depleting self-control will decrease the proportion of participants who will try the - for them - novel behavioral option of using a lifestyle NE substance.

## Methods

### Sample, treatment, procedure

One hundred and eighty-seven undergraduate sports students participated in return for course credit. After having read about lifestyle drug, prescription drug and illicit substance NE all of them were asked the question ‘Have you ever tried neuroenhancement i.e. used a substance with the explicit goal of enhancing your cognitive performance?’ Fifty-seven students who had tried NE were excluded from the experiment, in order to avoid including participants with a dominant response of neuroenhancing. The remaining 130 students (43% female, mean age = 22.76 ± 4.15 years) were seated in front of a computer monitor. All instructions were displayed on this monitor without further comment.

The participants began by completing an online questionnaire on trait self-control. Trait self-control is known to account for variance in behaviors that require self-control such as restraint eating or alcohol abuse [22]. Participants were randomly assigned to one of two conditions. The *non-depletion* group was asked to transcribe text to a sheet as quickly and accurately as possible for six minutes; the *depletion* group received the same instructions but was additionally required to omit all instances of the letters “e” and “n”. This task has been shown efficiently to induce ego-depletion in other experimental studies [23]. A manipulation check was performed afterwards. Participants were then told that they would be performing a similar concentration task a few minutes later. At the same time they were pointed to the opportunity that they could try to enhance their performance in the following task, by consuming one of the “energy sticks” from the package standing next to the computer monitor. A few moments later, when the participants had decided to progress with the experiment by clicking a button on the computer monitor, the experiment stopped, and the participants were debriefed and informed that no further test was required.

The *energy stick* is an ordinary over-the-counter product, a granulate containing 45 mg caffeine, 200 mg taurine and 5.85 mg vitamin B. This information and, most importantly, the energy sticks’ designated purpose - increasing cognitive performance - was flashily printed on the wrapping. The energy stick was chosen for the experiment because all the information apparent to the user is conducive to the notion that the product is able to improve cognitive performance (hence the energy stick meets the assumed functionality criterion for a neuroenhancer). In fact, there is less caffeine in this product than in one cup of coffee. The doses of both other ingredients are also unlikely to have any cognitive enhancing effects [24]. The conceptual congruence of the energy stick with NE was empirically confirmed in a pre-study (results available from the authors).

Experimental deception was in line with standard 8.07 of the APA Ethical Principles of Psychologists and Code of Conduct [25]. The University of Potsdam granted ethical approval for the study.

### Measures

Trait self-control was tested with a validated 25-item questionnaire [26]. An example item is ‘I find it difficult to control my needs’. Answers had to be given on a 4-point Likert-type scale, ranging from 1: ‘hardly ever’ to 4: ‘most of the time’. The internal consistency for this self-control scale was Cronbach’s α = .90 in our sample.

The manipulation check consisted of three questions on task difficulty (‘How difficult was this concentration task for you?’), mental exhaustion (‘How mentally exhausting was this concentration task for you?’), and motivation (‘How motivated were you to work on the concentration task as fast as possible?’). Participants had to indicate their answers on a 7-point Likert-type scale, ranging from ‘not at all’ to ‘extremely’.

Taking the energy stick (0 = No, 1 = Yes) served as the dependent variable. As participants were left unobserved during the experiment, this measure was recorded by counting the number of energy sticks after the participant had left the laboratory.

### Statistical analysis

To test our hypothesis, we conducted one stepwise logistic regression analysis with taking the energy stick as the dependent variable. After having controlled for trait self-control in step 1, the experimental factor (depletion: yes/no) was added in step 2.

## Results

### Randomization check

The two groups did not differ with respect to age, *t*(82) = .06, *p* = .95, or gender, *χ*^2^(2, 84) = 3.05, *p* = .22. Missing values in the assessment of age and gender may be due to the social sensitivity of NE, as participants were explicitly offered the option of declining to provide this information in order to protect their anonymity. There was a small, but statistically significant difference between the groups in trait self-control, *t*(128) = 2.13, *p* = .04, *d* = 0.38.

### Manipulation check

Participants in the depletion condition rated the transcription task as more difficult, *t*(128) = −5.73, *p* < .01, *d* = −1.01, and more mentally exhausting, *t*(128) = −3.57, *p* < .01, *d* = −0.63. Motivation did not differ between groups, *t*(128) = −0.19, *p* = .85. This confirmed the demanding nature of the chosen depletion task and ruled out the possibility that participants were differently motivated to solve the task in the two experimental conditions.

### Main analysis

**Figure 1:**
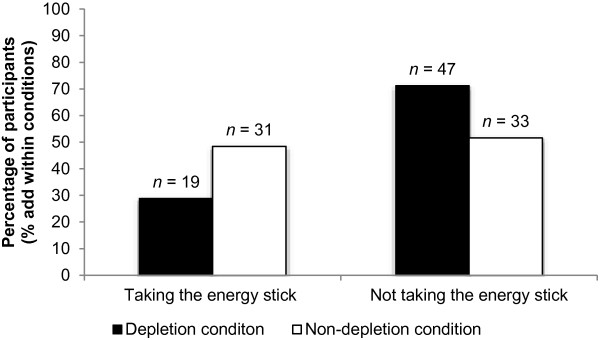
More than two-thirds (71%) of participants refrained from taking the energy stick in a state of mental exhaustion (ego-depletion), whereas half (48%) of the non-depleted participants chose to try this behavioral option.

Descriptive statistics are summarized in Table 1. The main result is illustrated in Figure 1. After having controlled for trait self-control (*OR* = 0.52, *p* = .19) in step 1, depletion explained incremental variance in the probability of taking the energy stick in step 2, *OR* = 0.37, *p* = .01, *CI95* = 0.17 - 0.79. Supporting our hypothesis, the relative probability of depleted participants choosing to take the energy stick was almost three times lower than in the control group.

**Table 1:** Descriptive statistics for experimental conditions

	***N***	**Age**^**a**^		**Gender %**^**a**^		**Self-control**		**NE intake** ***f***_***o***_
		**Mean**	***SD***	**Male**	**Female**	**Mean**	***SD***	***N***
Depletion	66	22.34	5.20	45.00	55.00	76.55	9.38	19
Non-depletion	64	22.28	4.26	60.46	39.54	79.94	8.72	31

*Note*. ^a^Numbers of age and gender might not add to 100% due to missing values, probably resulting as consequence of the social sensitivity of NE intake reporting.

## Discussion

Whereas almost half (48%) of our non-depleted participants chose to take the energy stick, 71% refrained from trying NE in a state of mental exhaustion (i.e. ego depletion). These findings are consistent with the strength model of self-control. Ego-depleted participants, who were unfamiliar with NE, failed to self-regulate. They were experimentally deprived of the mental resources necessary to decide in favor of the unfamiliar behavioral option of trying NE. The opportunity for self-regulation was however available to the non-depleted participants. Interpreted in the light of the strength model of self-control, it is possible that these participants took the energy stick as a means of self-regulating; perhaps in order to prepare themselves better for the anticipated cognitively demanding task.

Self-regulatory processes led half of the non-depleted participants (52%) to the decision not to try NE. Some of our participants might have seen NE as only one among several other, and perhaps better ways to prepare for the anticipated task. It may be that their weighing of subjective pros and cons - possible risks or side effects vs. the claimed benefit of improved cognitive performance in an experimental task - did not lead them to abandon their dominant response of not consuming a substance for cognitive enhancement. Pre-existing negative attitudes or beliefs about using NE substances, or a lack of belief that the energy stick really would improve or maintain their performance may have been critical to their decision.

Of course our result is a statistical one. Significantly different proportions of participants in the two conditions (ego-depletion vs. non-depletion) opted to accept or decline the opportunity to try NE as a means of improving cognitive performance. The results from a psychological experiment on human behavior will rarely lead to predictions accounting for the behavior of each and every individual, under all circumstances. But the results of our experiment provide evidence to support the hypothesis that the decision of students who have not yet tried NE as a means of improving cognitive performance might be informed by deliberate mental processes in a state of high self-control, rather than being an “act of weakness” or a simple stress reaction. Not until NE has become a dominant response, following reinforcing experiences of taking such substances, is it likely that the known negative aspects of ego-depletion would prompt an individual to carry out NE [19,20].

Our experiment is one of the few studies to have highlighted the positive preventative effects of ego-depletion - at least for those who think that NE is an undesirable behavior [27]. It is important to emphasize that this positive effect applies only to individuals, undergraduate university students in our sample, who have not yet had experience of using substances for cognitive performance enhancement. For individuals who have already consumed substances for this purpose, as a dominant response to stress for example, the state of ego-depletion might increase the probability of substance ingestion. Although this is what would be predicted by self-regulation theory, it has yet to be tested empirically as this study concentrated exclusively on people without NE experience.

Interpersonal differences in familiarity with NE have so far been neglected in the literature on the stress-neuroenhancement relationship [5]. Controlling for them might lead to a clearer pattern of correlations between these variables in epidemiological studies, and, most importantly, to the design of preventative or educational measures addressing NE in students or other groups.

### Limitations

Our sample consisted of sports students. This group may be disproportionately receptive to NE [1]. While the general readiness to engage in NE might be higher for these students, we can not think of a reason for a divergent psychological process to operate in the decision to try NE in this group. Nonetheless future studies should test the generalizability of our results to other samples. Further NE studies should vary 1) the time lag and 2) the frequency with which the substance is offered to participants. When self-control is re-established, do previously depleted participants still refrain from trying NE? After how many repeated instances of NE might this behavior become a dominant response, and hence become more likely to occur in the state of ego-depletion? Comparing our results with those from subjects who already make use of NE is a necessary next step. With this in mind, future experiments should focus on the role ego-depletion related passivity may play in the decision to use or abstain from NE [19]. Interactions with pre-existing attitudes or beliefs about NE, which have been shown to predict NE in students should also be analyzed [5].

### Implications

This is the first report of an experimental study of NE behavior. It extends research on NE by drawing on the theoretical perspective of the strength model of self-control, i.e. on different states of minds, when individuals are more or less likely to engage in NE. Our study may thus encourage other researchers to investigate further the possibility that the psychological state of ego-depletion may be followed by positive behavioral consequences for at least subgroups of individuals [27].

This study has implications for NE prevention policy. Undergraduates seem to decide to try NE for the first-time when sufficient cognitive resources are available. For inexperienced users NE might subjectively qualify as a reasonable thing to do. Psychological theory suggests that after reinforcing experiences NE can develop into a dominant behavioral response.

We therefore recommend that primary and secondary prevention efforts focus on providing information or even education about the possible negative health outcomes of NE use. The health-threatening effects associated with the chronic use of over-the-counter NE products (e.g. energy drinks) appear to be largely ignored by public opinion [28,29]. In addition, for those who are already habitual users of NE, alternative ways of coping with high demands should be contrasted with the NE option. At the individual level the workloads of some of our universities’ degree programs should be reconsidered in order to readjust some of the especially pressing demands in university students’ everyday environments. In our view, a strategy of *targeting interventions* at inter-individual differences is the most rational way of addressing NE as societal phenomenon [30,31]. Broader perspectives on NE should aim at the promotion of alternative methods of improving cognitive performance which would enhance the general welfare of a population [32].

## Competing interests

The authors declare that they have no competing interests.

## Authors’ contributions

FB, WW and RB designed the study. FB and WW conducted the statistical calculations and wrote the first draft of the manuscript. RB revised the first draft. All three authors then jointly worked on all subsequent versions of the manuscript. All three authors read and approved the final manuscript.
